# Impact of Plant Growth Promoting Bacteria on *Salicornia ramosissima* Ecophysiology and Heavy Metal Phytoremediation Capacity in Estuarine Soils

**DOI:** 10.3389/fmicb.2020.553018

**Published:** 2020-09-17

**Authors:** Jennifer Mesa-Marín, Jesús A. Pérez-Romero, Susana Redondo-Gómez, Eloísa Pajuelo, Ignacio D. Rodríguez-Llorente, Enrique Mateos-Naranjo

**Affiliations:** ^1^Departamento de Biología Molecular y Bioquímica, Facultad de Ciencias, Universidad de Málaga, Málaga, Spain; ^2^Departamento de Biología Vegetal y Ecología, Facultad de Biología, Universidad de Sevilla, Seville, Spain; ^3^Departamento de Microbiología y Parasitología, Facultad de Farmacia, Universidad de Sevilla, Seville, Spain

**Keywords:** bioaugmentation, halophyte, Odiel, Piedras, photosynthesis, growth, water, electron transport

## Abstract

*Salicornia ramosissima* is a C_3_ halophyte that grows naturally in South Western Spain salt marshes, under soil salinity and heavy metal pollution (mostly Cu, Zn, As, and Pb) caused by both natural and anthropogenic pressure. However, very few works have reported the phytoremediation potential of *S. ramosissima*. In this work, we studied a microbe-assisted phytoremediation strategy under greenhouse conditions. We inoculated plant growth promoting (PGP) and heavy metal resistant bacteria in pots with *S. ramosissima* and natural non-polluted and polluted sediments collected from Spanish estuaries. Then, we analyzed plant ecophysiological and metal phytoaccumulation response. Our data suggested that inoculation in polluted sediments improved *S. ramosissima* plant growth in terms of relative growth rate (RGR) (32%) and number of new branches (61%). *S. ramosissima* photosynthetic fitness was affected by heavy metal presence in soil, but bacteria inoculation improved the photochemical apparatus integrity and functionality, as reflected by increments in net photosynthetic rate (21%), functionality of PSII (F_*m*_ and F_*v*_/F_*m*_) and electron transport rate, according to OJIP derived parameters. Beneficial effect of bacteria in polluted sediments was also observed by augmentation of intrinsic water use efficiency (28%) and slightly water content (2%) in inoculated *S. ramosissima*. Finally, our results demonstrated that *S. ramosissima* was able to accumulate great concentrations of heavy metals, mostly at root level, up to 200 mg Kg^–1^ arsenic, 0.50 mg Kg^–1^ cadmium, 400 mg Kg^–1^ copper, 25 mg Kg^–1^ nickel, 300 mg Kg^–1^ lead, and 300 mg Kg^–1^ zinc. Bioaugmentation incremented *S. ramosissima* heavy metal phytoremediation potential due to plant biomass increment, which enabled a greater accumulation capacity. Thus, our results suggest the potential use of heavy metal resistant PGPB to ameliorate the capacity of *S. ramosissima* as candidate for phytoremediation of salty polluted ecosystems.

## Introduction

Environmental pollution has become a major public concern over the last century. Specially, heavy metal content in water and soil has increased due to rapid worldwide industrial development (Usman et al., 2018). This problem is further compounded by soils simultaneously affected by salinity because of climate change and irrigated agriculture (Liang et al., 2017). To overcome this, phytoremediation has been largely studied and suggested as an environmentally friendly and low-cost clean-up method. However, a great proportion of the known phytoremediators are glycophytes, and they cannot survive the combination of salt and heavy metal pollution long enough to be effective (Wang et al., 2014). Conversely, halophytes are able to survive in saline environments (Flowers and Colmer, 2008) and are naturally adapted to tolerate metals, compared to glycophytes (Van Oosten and Maggio, 2015; Liang et al., 2017). Thus, halophytes may represent excellent candidates for phytoremediation of heavy metals in soils with high salinity (Anjum et al., 2014), as have been demonstrated by several species from the genera *Atriplex, Tamarix, Sporobolus, Juncus, Suaeda*, etc. (Manousaki and Kalogerakis, 2011; Redondo-Gómez, 2013). Among processes that halophytes use for phytoremediation are: (i) phytoextraction (plants take metals from the soil and transport and concentrate them in harvestable above-ground tissues), (ii) phytovolatilization (plants take water soluble metals and release them as they transpire the water), (iii) phytostabilization (plants completely immobilize the metals through accumulation by roots or precipitation within the rhizosphere), (iv) rhizofiltration (plants absorb, concentrate and precipitate pollutants of water), (v) phytoaccumulation (pollutants are accumulated in plants’ biomass), and (vi) phytodegradation (pollutants are degraded into insoluble or non-toxic compounds) (Anjum et al., 2014). Phytoremediation strategy is based on major plants, but also on associated microbes and their processes. In this sense, plant growth promoting bacteria (PGPB) bioaugmentation has been proposed as a strategy to improve innate heavy metal phytoremediation capacity in halophytes, since it has been proven to ameliorate plant growth, stress tolerance and phytoremediation potential (Backer et al., 2018). Indeed, positive results have been recently obtained for *Spartina, Arthrocnemum*, and *Suaeda* species, which incremented their root metal phytoaccumulation capacity when they were PGPB-treated (Mateos-Naranjo et al., 2015; Mesa et al., 2015b; Navarro-Torre et al., 2017a; Gómez-Garrido et al., 2018).

An ideal case of study in this matter can be found in Odiel salt marsh (SW Spain). In this area, halophytes grow in soils containing salt and heavy metals. Recent studies in Odiel soils registered around 200 mM NaCl and approximately 150 mg Kg^–1^ As, 3 mg Kg^–1^ Cd, 5 mg Kg^–1^ Co, 900 mg Kg^–1^ Cu, 30 mg Kg^–1^ Ni, 300 mg Kg^–1^ Pb, and 1700 mg Kg^–1^ Zn (Mesa et al., 2016). Pollution levels in the estuary are caused by both natural and anthropogenic pressure (Mesa et al., 2016). *Salicornia ramosissima* J. Woods is a C_3_ halophyte that grows naturally in Odiel estuary area. It is a widespread plant on the European coastline, including salt marshes of the Iberian Peninsula, where it usually occupies the higher reaches of the salt marsh and is a pioneer species in the colonization of the intertidal zones of such habitats (Davy et al., 2001). These features, together with the potential for metal bioremediation of the genus *Salicornia* observed by some authors (Ozawa et al., 2010; Sharma et al., 2010; Kaviani et al., 2017a,b; Lou et al., 2020), make *S. ramosissima* an excellent candidate for land restoration. For example, root exudates from *Salicornia europaea* could form Pb-complexes which help Pb-stabilization and, therefore, remediation (Pan et al., 2012). Also, Shrestha et al. (2006) reported in *Salicornia bigelovii* 2.2-fold more biogenic volatile Se compared to the control. However, the metal accumulation ability of the species *S. ramosissima* has been scarcely studied. Only a few works reported *S. ramosissima* phytoaccumulation abilities. Pedro et al. (2013) concluded that this species may be useful for phytoaccumulation and phytostabilization, since plants have a considerable bioaccumulation potential and were able to bioaccumulate Cd mainly in the roots, acting like a sink for this metal and preventing it from becoming available to other organisms. Pérez-Romero et al. (2016) confirmed the traits of Cd accumulation and tolerance of *S. ramosissima*, and stated that this tolerance could be due to many of essential steps of its photosynthetic pathway were tolerant to Cd excess. On the other hand, PGPB treatments have been never tested on *S. ramosissima* with phytoremediation purposes. In a previous work, we isolated PGPB from the rhizosphere of *S. ramosissima* growing in SW Spain salt marshes and designed a bacterial consortium for inoculation (Mesa-Marín et al., 2019c). We hypothesize that *S. ramosissima* may accumulate heavy metals in its tissues when it grows in a polluted soil, and that bioaugmentation treatment with the bacterial inoculum mentioned above may improve growth of *S. ramosissima* and its metal accumulation capacity. Thus, this is the first work that studies the phytoremediation capacity of PGPB-treated *S. ramosissima*.

Then, this work aimed at (1) assessing the tolerance to heavy metals of the bacteria selected for inoculation, in order to ensure they can be used in polluted sediments, (2) describing heavy metal accumulation capacity of *S. ramosissima* growing in natural salt marsh sediments, and (3) analyzing *S. ramosissima* response to PGPB inoculation in terms of growth, photosynthetic fitness, and metal accumulation.

## Materials and Methods

### Plant and Soil Source

Seeds of *S. ramosissima* were collected in December 2017 from Odiel (37°15′N, 6°58′W; SW Spain) and Piedras (37°16′09.1′′N 7°09′36.4′′W; SW Spain) marshes. Later, they were kept in darkness at 4°C up to 3 months. Prior to the experiment, seeds were surface-disinfected and germinated as indicated in Mateos-Naranjo et al. (2015). Seedlings were planted in perlite (in 11 cm diameter × 9 cm high pots), in a glasshouse with 40–60% relative humidity, natural daylight and 21–25°C. 20% Hoagland’s solution (Hoagland and Arnon, 1938) was used to irrigate pots as necessary. For our experiment, two natural marshy soils were used. Soil from the Piedras estuary (Huelva, SW Spain), with no anthropogenic influence and thereby non-polluted, was used to grow *S. ramosissima* control plants, and soil from the Odiel estuary (Huelva, SW Spain) was used to grow plants under polluted conditions. Metal concentration of both soils is available in Table 1. Arsenic, copper and lead concentrations were especially high in Odiel salt marshes (ca. 800, 1000, and 1100 mg Kg^–1^, respectively), constituting 60×, 30×, and 35× times, respectively, compared to Piedras sediments.

**TABLE 1:** Metal(loid) concentrations in mg Kg^–1^ in sediments from Odiel to Piedras salt marshes used in this study.

Location	As	Cd	Cu	Ni	Pb	Zn
Piedras	14.22 ± 0.41	0.20 ± 0.12	37.35 ± 1.43	24.28 ± 0.47	31.95 ± 0.16	108.57 ± 3.75
Odiel	853.54 ± 34.45	2.19 ± 0.7	1076.39 ± 58.55	28.77 ± 1.48	1139.85 ± 58.76	809.92 ± 40.06

*Values are means ± S.E.*

### Bacterial Tolerance Against Heavy Metals

Bacteria used as an inoculum in this experiment were isolated from *S. ramosissima* rhizosphere, growing in the Tinto salt marsh (SW Spain, 37° 13′ 51.40′′ N 6° 54′ 28.40′′ W) (Mesa-Marín et al., 2019c). They were identified as *Vibrio neocaledonicus* SRT1, *Thalassospira australica* SRT8 and *Pseudarthrobacter oxydans* SRT15, and they were selected among other rhizosphere isolates based on their outstanding Plant Growth Promoting (PGP) properties (Mesa-Marín et al., 2019c). This bacterial consortium showed nitrogen fixation abilities, phosphate solubilization and biofilm-forming capacity, as well as production of 1-aminocyclopropane-1-carboxylate (ACC) deaminase, indole-3-acetic acid (IAA), and siderophores (Table 2; Mesa-Marín et al., 2019c). For this work, heavy metal tolerance of these bacteria was analyzed. Single strains were streaked on TSA 0.2 M NaCl medium (in line with salt marshes soil conductivity) and supplemented with increasing concentrations of heavy metals and metalloids from stock solutions: NaAsO_2_ 0.5 M, CdCl_2_ 1 M, CuSO_4_ 1 M, CoCl_2_ 1 M, NiCl_2_ 0.2 M, ZnSO_4_ 1 M, and Pb(NO_3_)_2_ 0.5 M (in order to avoid Pb precipitation when mixing with TSA, the same concentration of EDTA needs to be added to the plates). Tolerance was assessed for each strain and heavy metal separately. After incubation at 28°C during 72 h, maximum tolerable concentration (MTC) was expressed for each strain and element, namely the maximum concentration of a metal or metalloid that permitted bacterial growth in plates.

**TABLE 2:** Plant growth promoting traits for bacterial consortia used in this study (Mesa-Marín et al., 2019c).

Strain	Nitrogen fixation^*a*^	Phosphate solubilization^*b*^	Siderophores production^*b*^	IAA production (mg/mL)	Biofilm production^*a*^	ACC deaminase activity (μ mol α -cetog h^–^^1^ mg prot^–^^1^)
SRT1	+	10	20	5.65	+	−
SRT8	−	−	−	−	+−	1.24
SRT15	+	9	−	20.99	+	−

*^*a*^ (+) presence or (−) absence of growth; ^*b*^ halo diameter in mm.*

### Experimental Treatments

Pots containing Piedras and Odiel sediment were planted with *S. ramosissima* and assigned randomly to two bacterial bioaugmentation treatments: non-inoculated (control) and inoculated with the selected PGPB (*n* = 40, 2 soils × 2 bacterial treatments = 4 treatments, 10 pots per treatment). Pots were put in trays with tap water down to a depth of 1 cm. During the experimental period, 30 days, water level in the trays were checked every 2 days. Inoculated pots were watered with 50 ml of a bacterial suspension at the beginning of the experiment, while control pots were watered with 50 ml of tap water. Inoculant suspensions were prepared as described in Mesa-Marín et al. (2019c). Finally, bacteria pellets were resuspended in tap water until it was obtained a suspension with OD_600_ = 1 (approx. 10^8^ cells per ml) for each strain. Equal amounts of each strain were mixed to obtain the final OD_600_ = 1 inoculant suspension. The final volume of inoculant suspension was 1 L, to water 20 pots with 50 ml each.

### *Salicornia ramosissima* Growth and Water Status Analysis

Six *S. ramosissima* plants were harvested before the initiation of the experiment and the rest were collected after 30 days of bioaugmentation treatment. The relative growth rate (RGR) of *S. ramosissima* was calculated (Mesa et al., 2015b). Also, all *S. ramosissima* height and number of ramifications were recorded at the beginning and the end of the experimental period. Furthermore, water content (WC) of primary branches (*n* = 9 per each soil and bacterial treatment combination) were calculated as follow:

WC = [(FW – DW)/FW] × 100

where FW = fresh weight of the branches and DW = dry weight after oven-drying at 80°C for 48 h.

### *Salicornia ramosissima* Photosynthetic Performance Analysis

One day before plants were harvested, gas exchange parameters were measured on primary branches (*n* = 10, per each soil and bacterial treatment combination) using an infrared gas analyzer (LI-6400, LI-COR Inc., Neb., United States) in an open system. It was equipped with a light leaf chamber (Li-6400-02B, Li-Cor Inc.). Thus, stomatal conductance (g_*s*_), intercellular CO_2_ concentration (C_*i*_), net photosynthetic rate (A_*N*_), and instantaneous water use efficiency (_*i*_WUE; ratio between A_*N*_ and g_*s*_) were obtained with the following leaf chamber settings: photon flux density (PPFD) of 1000 μmol photons m^–2^ s^–1^ (with 15% blue light to maximize stomatal aperture), CO_2_ concentration (C_*a*_) surrounding leaf of 400 μmol mol^–1^ air, air temperature of 24 ± 1°C, relative humidity of 45 ± 5%, and vapor pressure deficit of 2.0–3.0 kPa.

Furthermore, chlorophyll fluorescence measurements were performed in the same branches of gas exchange analysis using a FluorPen FP100 (Photo System Instruments, Czechia). Thus, the chlorophyll *a* fast kinetics, or JIP-test (or Kautsky curves), which depicts the rate of reduction kinetics of various components of PSII, were measured in 30 min dark-adapted branches (*n* = 6, per each soil and bacterial treatment combination), using the pre-programmed OJIP test implemented in the pre-programmed protocols of the FluorPen. Maximum quantum efficiency of PSII photochemistry (F_*v*_/F_*m*_), absorbed energy flux (ABS/CS), trapped energy flux (TR/CS), electron transport energy flux (ET/CS), and dissipated energy flux (DI/CS) per reaction center derived for OJIP were calculated (Strasser et al., 2004).

### Chemical Analyses of *Salicornia ramosissima* Tissues

*Salicornia ramosissima* roots and leaves were carefully washed with distilled water at the end of the experiment to eliminate ions from their surface before the analysis. At the end of the experiment, dried roots and leaves were ground (Mateos-Naranjo et al., 2008). 0.5 g sub-samples were taken from the roots and the leaves of the ten replicate plants. Phosphorus (P), sodium (Na), magnesium (Mg), manganese (Mn), calcium (Ca), potassium (K), arsenic (As), cadmium (Cd), copper (Cu), nickel (Ni), lead (Pb), and zinc (Zn) were measured by inductively coupled plasma (ICP) spectroscopy (ARL-Fison 3410, United States) in tissues and soil.

### Statistical Analysis

Statistics were analyzed with “Statistica” v. 10.0 (StatSoft Inc.). Generalized linear models (GLM) aided to analyze the interactive effects of soil pollution and bacterial treatments (as categorical factors) on the growth, chlorophyll fluorescence and gas exchange of *S. ramosissima* plants (as dependent variables), followed by a LSD (*post hoc*) test for multiple comparisons analysis. Nutrient and heavy metal content in *S. ramosissima* leaves and roots after different bioaugmentation treatments were compared by the Student test (*T*-test), and values were analyzed for each tissue by using one-way ANOVA (*F*-test). Data normality was tested with the Kolmogorov–Smirnov test and data homogeneity of variance with the Brown–Forsythe test. Tukey (*post hoc*) tests were used for identification of important contrasts. In all cases, a significance level of *p* < 0.05 was used.

## Results

### Rhizobacteria Tolerance to Heavy Metals

Bacteria selected in this work for bioaugmentation treatments were *Vibrio neocaledonicus* SRT1, *Thalassospira australica* SRT8, and *Pseudarthrobacter oxydans* SRT15. They were isolated from *S. ramosissima* rhizosphere growing in heavy metal polluted Tinto salt marsh (SW Spain). Isolation was conducted by pour-plating a mix of rhizospheric soil and physiological saline solution (NaCl 0.9% w/v) on tryptic soy agar (TSA) NaCl 0.2 M and further streaking of single colonies in the same medium, with incubations at 28°C for 24–48 h (Mesa-Marín et al., 2019c). SRT1 exhibited nitrogen fixation and phosphate solubilization, as well as the capacity to produce siderophores and biofilms. SRT8 produced ACC deaminase and SRT15 showed the best auxins production (Table 2). In this work, heavy metal tolerance of these strains was tested (Table 3). Strain SRT1 showed the broadest tolerance, with particular emphasis on As, Cd, and Co. Overall, the three strains showed a notable tolerance to Zn (ranging from 1 to 3 mM), Pb (from 3 to 5 mM), Ni (up to 9 mM), Cu (up to 3 mM), and Co (from 3 to 13 mM).

**TABLE 3:** Maximum tolerable concentration (MTC) in mmoles L^–1^ (mM) for each rhizobacteria strain and metal(loid) tested.

Strain	As (NaAsO_2_)	Cd (CdCl_2_)	Co (CoCl_2_)	Cu (CuSO_4_)	Ni (NiCl_2_)	Pb (Pb(NO_3_)_2_)	Zn (ZnSO_4_)
SRT1	5	4	13	3	9	5	3
SRT8	<1	0.3	3	3	1	5	1
SRT15	<1	<0.1	5	1	9	3	1

### Soil Bioaugmentation Effect on *Salicornia ramosissima* Growth and Water Content

**FIGURE 1:**
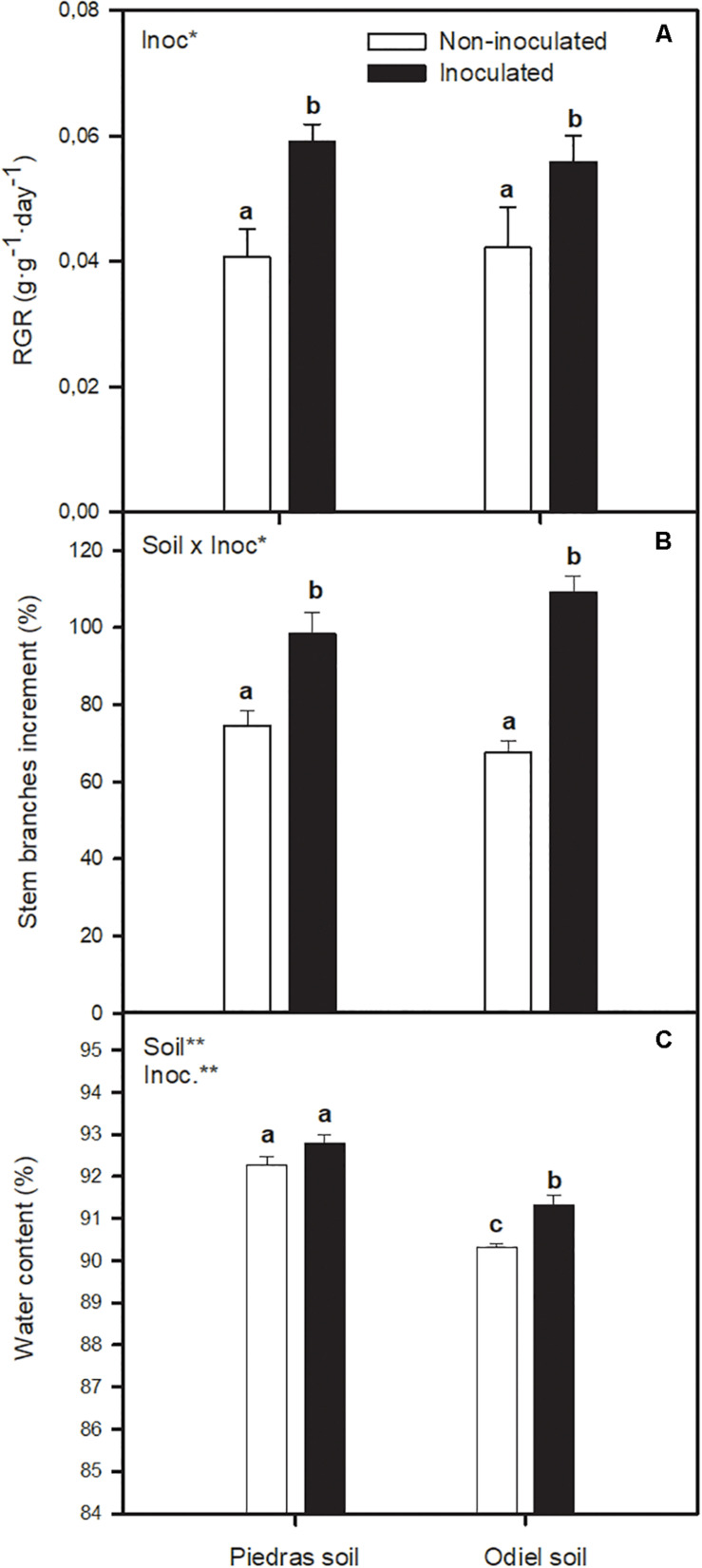
Effect of soil bioaugmentation treatments (non-inoculated and inoculated) with a bacterial consortium integrated by *Vibrio neocaledonicus* SRT1, *Thalassospira australica* SRT8 and *Pseudarthrobacter oxydans* SRT15 on **(A)** relative growth rate, RGR, **(B)** number of stem branches and **(C)** water content of *Salicornia ramosissima* plants grown in natural soil from non-polluted Piedras and polluted Odiel marshes for 30 days. Values are means ± S.E. (*n* = 10). “Inoc” or “Soil” in the upper left corner of the panel indicates main or interaction significant effect (*p < 0.1, **p < 0.01). Different letters indicate means that are significantly different from each other (*p* < 0.05).

After 30 days of treatment bacterial inoculation improved *S. ramosissima* RGR compared to non-inoculated plants (Figure 1A). Soil bioaugmentation incremented RGR 45% in non-polluted Piedras sediments and 32% in polluted Odiel sediments (GLM_*inoc*_, *p* < 0.05). Also, at the end of the treatment, inoculated *S. ramosissima* showed significant increments of stem branches compared to control (Figure 1B), 32% in non-polluted sediments and 61% in polluted sediments (GLM_*soil x inoc*_, *p* < 0.05). However, for *S. ramosissima* stem height there was no statistically significant difference in any case (data not shown). Plant water content (Figure 1C) did not vary with inoculation in non-polluted sediments. Conversely, it was higher in inoculated plants grown in polluted sediments (GLM_*soil, inoc*_ *p* < 0.05).

### Soil Bioaugmentation Effect on *Salicornia ramosissima* Photosynthetic Apparatus Performance

**FIGURE 2:**
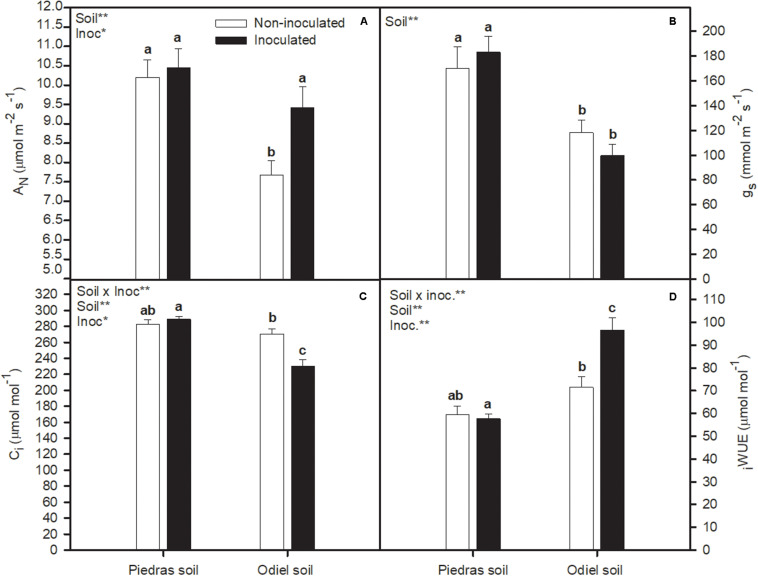
Effect of soil bioaugmentation on *S. ramosissima* **(A)** net photosynthetic rate, A_*N*_, **(B)** stomatal conductance, g_*s*_, **(C)** intercellular CO_2_ concentration, C_*i*_, and **(D)** instantaneous water-use efficiency, _*i*_WUE, after 30 days of treatment. Values are means ± S.E. (*n* = 10). “Inoc” or “Soil” in the upper left corner of the panel indicates main or interaction significant effect (*p < 0.1, **p < 0.01). Different letters indicate means that are significantly different from each other (*p* < 0.05).

**FIGURE 3:**
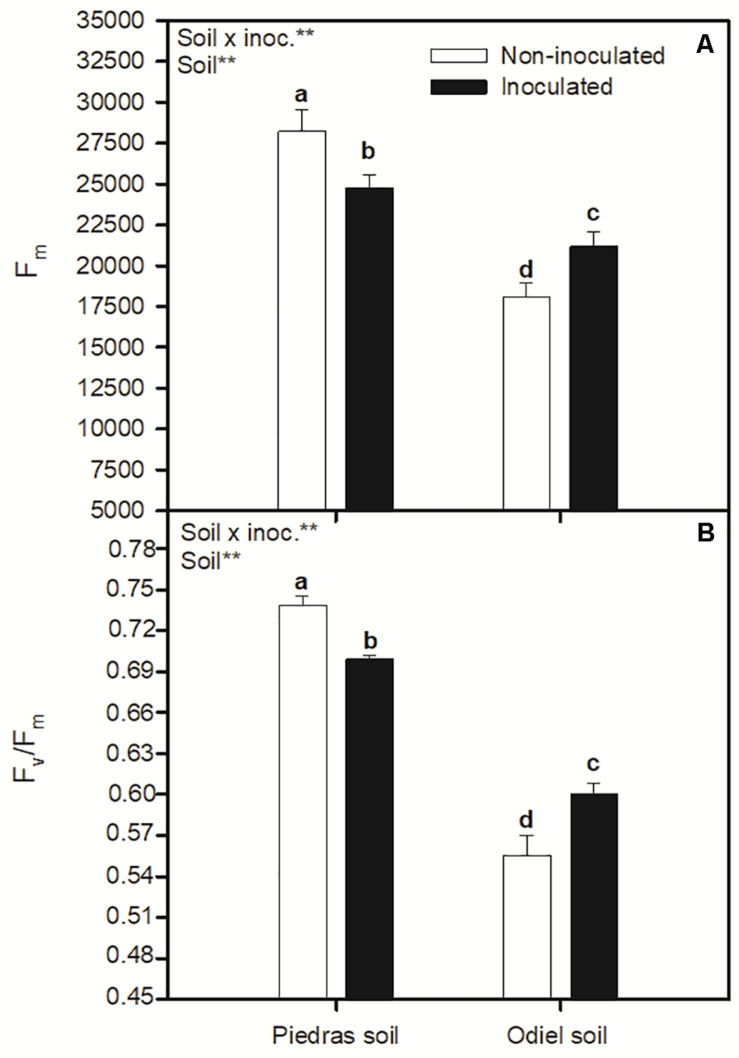
Effect of soil bioaugmentation on *S. ramosissima* **(A)** maximal fluorescence level in the dark-adapted State, F_*m*_, and **(B)** maximum quantum efficiency of PSII photochemistry, F_*v*_/F_*m*,_ after 30 days of treatment. Values are means ± S.E. (*n* = 10). “Inoc” or “Soil” in the upper left corner of the panel indicates main or interaction significant effect (**p < 0.01). Different letters indicate means that are significantly different from each other (*p* < 0.05).

**FIGURE 4:**
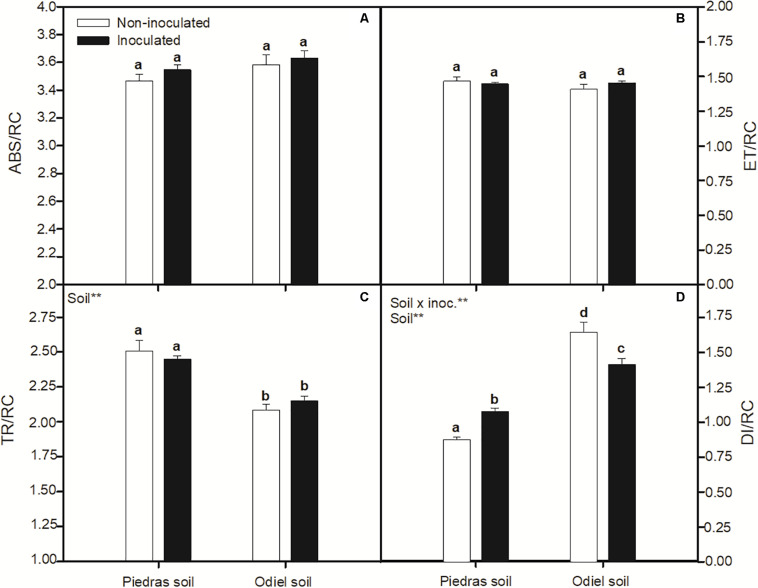
Effect of soil bioaugmentation on *S. ramosissima* **(A)** ABS/RC, **(B)** ET/RC, **(C)** TR/RC, and **(D)** DI/RC after 30 days of treatment. Values are means ± S.E. (*n* = 10). “Inoc” or “Soil” in the upper left corner of the panel indicates main or interaction significant effect (**p < 0.01). Different letters indicate means that are significantly different from each other (*p* < 0.05).

As shown in Figure 2, *S. ramosissima* growing in polluted sediments had higher _*i*_WUE, and lower A_*N*_, g_*s*_, and C_*i*_, compared to plants growing in non-polluted sediments (*T*-test, *p* < 0.05). Soil bioaugmentation with rhizobia did not altered *S. ramosissima* A_*N*_, g_*s*_, C_*i*_, and _*i*_WUE in non-polluted soil (Figures 2A–D). Conversely, soil bioaugmentation in polluted sediments significantly incremented A_*N*_ and _*i*_WUE, while lowered C_*i*_ (GLM _*soil, inoc, soil* × *inoc*_ *p* < 0.05; Figures 2A–D). Figure 3 illustrates changes in chlorophyll fluorescence parameters after 30 days of treatments. In this case, F_*m*_ and F_*v*_/F_*m*_ showed a clear pattern of statistically significant variation in both sediments and inoculation treatments. Both parameters decreased for inoculated plants in non-polluted soil, while they increased after inoculation in polluted soils (GLM _*soil, soil* × *inoc*_, *p* > 0.05; Figures 3A,B). Finally, focusing on OJIP derived parameters (Figure 4), we found that bioaugmentation treatments and soil pollution degree did not affect ABS/CS and ET/CS values (GLM, *p* > 0.05; Figures 4A,B), while TR/C values decreased in polluted soil in similar degree at both inoculation treatments (GLM _*soil*_, *p* < 0.05; Figure 4C). Contrarily, there was an increment in DI/RC values under metal pollution conditions but this augmentation was more accused in non-inoculated plants (GLM _*soil* × *inoc*_, *p* > 0.05; Figure 4D).

### Soil Bioaugmentation Effect on *Salicornia ramosissima* Tissues Nutrient Profile

At the end of the experiment, rhizobacterial consortium addition altered nutrient profile in a greater extent in leaves than in roots of *S. ramosissima* (two-way Anova, *p* < 0.01; Table 4). Soil bioaugmentation in non-polluted sediments produced greater concentrations of Ca, Mg, Mn, and Na in *S. ramosissima* leaves than their non-inoculated counterparts. Conversely, soil bioaugmentation in polluted soil only increased Na concentrations in leaves (two-way Anova, *p* < 0.01; Table 4). It is noteworthy that rhizobacterial treatment increased in both cases Na uptake in leaves 16%.

**TABLE 4:** Total calcium (Ca), potassium (K), magnesium (Mg), manganese (Mn), sodium (Na), and phosphorous (P) concentrations for *Salicornia ramosissima* tillers and roots after 30 days of bioaugmentation treatment in non-polluted Piedras and polluted Odiel sediments.

Soil, tissue and treatment	Ca (mg g^–^^1^)	K (mg g^–^^1^)	Mg (mg g^–^^1^)	Mn (mg Kg^–^^1^)	Na (mg g^–^^1^)	P (mg g^–^^1^)
**Piedras**						
**Leaf**						
Control	0.34 ± 0.01^a^	1.42 ± 0.06^a^	0.53 ± 0.01^a^	141.16 ± 4.55^a^	11.03 ± 0.44^a^	0.23 ± 0.02^a^
Inoculated	0.43 ± 0.01^b^	1.45 ± 0.07^a^	0.64 ± 0.01^b^	174.80 ± 3.56^b^	13.18 ± 0.45^b^	0.24 ± 0.01^a^
**Root**						
Control	0.26 ± 0.02^a^	1.19 ± 0.08^a^	0.38 ± 0.02^a^	267.76 ± 1.27^a^	1.57 ± 0.09^a^	0.08 ± 0.00^a^
Inoculated	0.24 ± 0.01^a^	1.21 ± 0.05^a^	0.40 ± 0.02^a^	258.69 ± 4.91^a^	1.52 ± 0.06^a^	0.07 ± 0.00^a^
**Odiel**						
**Leaf**						
Control	0.31 ± 0.04^a^	1.28 ± 0.16^a^	0.39 ± 0.05^a^	67.57 ± 7.81^a^	9.35 ± 0.37^a^	0.25 ± 0.03^a^
Inoculated	0.38 ± 0.03^a^	1.28 ± 0.10^a^	0.45 ± 0.04^a^	71.28 ± 3.89^a^	11.44 ± 0.46^b^	0.27 ± 0.01^a^
**Root**						
Control	0.40 ± 0.04^a^	1.41 ± 0.01^a^	0.32 ± 0.01^a^	349.97 ± 13.82^a^	1.26 ± 0.06^a^	0.21 ± 0.01^a^
Inoculated	0.34 ± 0.03^a^	1.34 ± 0.05^a^	0.31 ± 0.01^a^	292.09 ± 5.06^b^	1.16 ± 0.02^a^	0.19 ± 0.00^a^

*Values are means ± SE. *n* = 3 and different letters indicate means that are significantly different from each other (*p* < 0.05).*

### *Salicornia ramosissima* Heavy Metal Accumulation Capacity and Effect of Soil Bioaugmentation

*Salicornia ramosissima* growing in polluted sediments accumulated great concentrations of heavy metals in its tissues, compared to plants growing in non-polluted sediments (Tables 4, 5). This natural bioaccumulation capacity was particularly noticeable in *S. ramosissima* roots, reaching concentrations of 31, 14 or 17 times more As, Cu, and Pb, respectively, than plants growing in non-polluted sediments (*T*-test, *p* < 0.01; Table 6). Ni concentration was similar in both cases, as Ni concentration in both soils was the same (Table 1).

**TABLE 5:** Metal(loid) concentrations in mg Kg^–1^ found in *S. ramosissima* leaves and roots growing in non-polluted Piedras sediments after 30 days of experiment.

Tissue	Treatment	As	Cd	Cu	Ni	Pb	Zn
Leaves	Non-inoculated	0.44 ± 0.17^a^	<0.01 ± 0.00^a^	8.08 ± 0.59^a^	0.43 ± 0.12^a^	0.73 ± 0.16^a^	18.56 ± 1.05^a^
	Inoculated	0.47 ± 0.13^a^	<0.01 ± 0.00^a^	9.46 ± 0.19^a^	1.07 ± 0.29^b^	0.99 ± 0.28^a^	26.30 ± 0.80^b^
Root	Non-inoculated	8.21 ± 0.48^a^	0.13 ± 0.10^a^	29.60 ± 1.07^a^	26.23 ± 2.27^a^	17.68 ± 1.01^a^	102.20 ± 3.98^a^
	Inoculated	10.73 ± 0.53^a^	0.23 ± 0.11^a^	34.38 ± 1.01^b^	33.44 ± 0.93^b^	21.72 ± 0.79^b^	99.47 ± 1.53^a^

*Values are means ± S.E. Different letters indicate statistical differences for each metal(loid) and tissue between bioaugmentation treatments.*

**TABLE 6:** Distribution of arsenic (As), cadmium (Cd), copper (Cu), nickel (Ni), lead (Pb), and zinc (Zn) in estuarine soils of our study and *S. ramosissima* in natural conditions (non-inoculated).

Odiel vs. Piedras	As	Cd	Cu	Ni	Pb	Zn
Soil	60	10	29	1	36	7
*S. ramosissima* roots	31	4	14	1	17	3
*S. ramosissima* leaves	18	1	2	1	14	2

*The table displays the times that every heavy metal is more concentrated in polluted Odiel sediments and *S. ramosissima* growing in them, compared to non-polluted Piedras sediments and *S. ramosissima* growing in them. For example, arsenic was 60 times more concentrated in Odiel than in Piedras soil, and *S. ramosissima* growing in Odiel soil accumulated 31 times more arsenic in roots and 18 in leaves than *S. ramosissima* growing in Piedras soil.*

**FIGURE 5:**
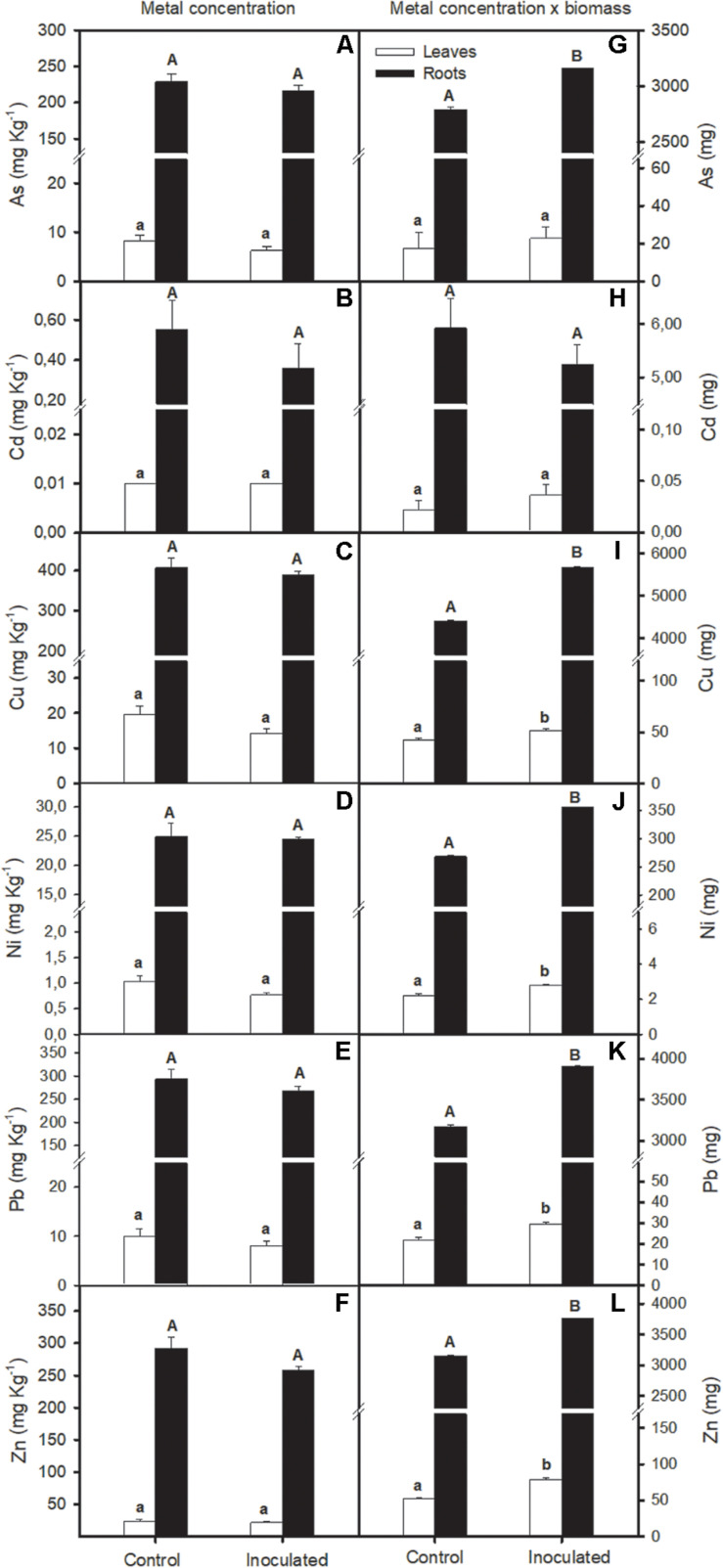
Effect of soil bioaugmentation in polluted Odiel sediments on *S. ramosissima* heavy metal accumulation after 30 days of treatment initiation. In the left column, total **(A)** arsenic, As, **(B)** cadmium, Cd, **(C)** copper, Cu, **(D)** nickel, Ni, **(E)** lead, Pb, and **(F)** zinc, Zn, for leaves and roots of *S. ramosissima*. In the right column, total concentration values have been normalized for *S. ramosissima* biomass after 30 days of treatment **(G–L)**. Values are means ± S.E. (*n* = 3). Different capital letters indicate statistical differences between inoculation treatments in roots and different lower case letters in leaves (*p* < 0.05).

Bioaugmentation effect on *S. ramosissima* metal accumulation capacity was studied in polluted sediments (Figure 5). Concentration of ions was higher in roots than in *S. ramosissima* leaves (*T*-test, *p* < 0.01; Figures 5A–L). Our data revealed that there was no statistical difference in metal concentration per Kg of *S. ramosissima* roots or leaves between inoculation conditions after 30 days of experiment (one-way ANOVA, *p* > 0.05; Figures 5A–F). However, when *S. ramosissima* biomass gaining was taken into account for each treatment at the end of the experiment, results varied. This is, *S. ramosissima* plants inoculated with the rhizobacterial consortium, which showed greater growth rates than the non-inoculated ones, showed higher total metal content than the control for all metals assayed, except for Cd (one-way ANOVA, *p* < 0.05; Figures 5G–L).

## Discussion

In this work, we used two sediments from SW Spain salt marshes where *S. ramosissima* grows naturally, with historically well-known different level of heavy metal pollution. Metal concentration in Piedras salt marsh, without anthropogenic influence, remains very similar to that reported in the last decade (Mesa et al., 2016). On the contrary, data obtained in Odiel salt marshes sediments revealed that the concentration of lead and arsenic has increased more than two-fold compared with data compiled in the last decade, while cadmium and zinc concentrations have decreased (Mesa et al., 2016). Spanish Government established threshold values for metal clean-up intervention in soil (de Andalucía, 1999). Concentration of As (ca. 800 mg Kg^–1^), Cu (ca. 1000 mg Kg^–1^), and Pb (ca. 1100 mg Kg^–1^) in Odiel sediments surpassed threshold values that Spanish Government stablished as intervention limits, this is, 100, 500, and 1000 mg Kg^–1^, respectively (de Andalucía, 1999). This fact highlights the importance of looking into efficient eco-friendly restoration strategies in these areas, which have a high ecological value. In this work, we studied a proposal of phytoremediation with PGPB-assisted *S. ramosissima*. This is in line with our previous studies on PGPB treatments with several *Spartina* genus in the same area (Mateos-Naranjo et al., 2015; Mesa et al., 2015b).

Data collected in our experiment showed that *S. ramosissima* responded to bioaugmentation with a bacterial consortium composed by *Vibrio neocaledonicus* SRT1, *Thalassospira australica* SRT8, and *Pseudarthrobacter oxydans* SRT15 (Mesa-Marín et al., 2019c), as could be seen from diverse physiological parameters. On one hand, PGPB bioaugmentation had in both non-polluted and polluted sediments a positive impact on *S. ramosissima* growth, reflected by RGR results and number of *S. ramosissima* stem branches. However, height of plants did not vary with bioaugmentation. Increased plant growth may be due to the well-known beneficial properties on plant development and stress tolerance of PGPB (Glick, 2012). In particular, the use of the three PGP strains mentioned above allowed *S. ramosissima* to benefit from six PGP properties: siderophores, IAA and ACC deaminase production, phosphates solubilization, biofilm formation and nitrogen fixation (Mesa-Marín et al., 2019c). IAA is a phytohormone with a crucial role in cell differentiation and division, and therefore in root development (Duca et al., 2014). Siderophores sequester and solubilize iron from the soil, facilitating to the plant enough acquisition of this element, especially when there are great amounts of other competing metals (Neilands, 1995). Also, bacterial phosphate solubilizing activity provides plants with a bioavailable form of phosphorus, from immobilized inorganic and organic P compounds that can be found in soil (Glick, 2012). Nitrogen fixing bacteria provide assimilable N to the plant (de Souza et al., 2015). Bacteria with the enzyme ACC deaminase degrade the plant ethylene precursor, diminishing ethylene levels in a stressed or developing plant, especially in the presence of salinity excess, and therefore, alleviating plant damage (Glick, 2014; Mesa-Marín et al., 2019b). Finally, there is evidence of the role of biofilms in the presence of heavy metals, by keeping them concentrated, partitioned and immobilized, minimizing the environmental hazards (Bogino et al., 2013). On the whole, *Vibrio neocaledonicus* SRT1, *Thalassospira australica* SRT8, and *Pseudarthrobacter oxydans* SRT15 may improve *S. ramosissima* nutrient acquisition and stress tolerance to heavy metals, which reflects in an increment in RGR and branch development. In our previous works with *Spartina* species growing in Piedras, Odiel, and Tinto sediments (SW Spain), *Spartina densiflora* and *Spartina maritima* also registered increments in their growth rates after PGPB bioaugmentation, in line with the obtained for *S. ramosissima* (Mateos-Naranjo et al., 2015; Mesa et al., 2015b).

On the other hand, the positive effect of bacteria inoculation was also supported by improvement in photosynthetic parameters of *S. ramosissima*. Overall, heavy metal pollution clearly affected *S. ramosissima* photosynthetic metabolism, as observed from lower net photosynthetic rate (A_*N*_) values in control plants growing in polluted sediments compared to non-polluted. In this case, unlike growth, PGPB bioaugmentation had only significant effect in polluted conditions. PGPB inoculation induced a higher plant photosynthetic performance, indicated by greater A_*N*_ values. Furthermore, the photosynthetic improvement in inoculated *S. ramosissima* may be related to the greater integrity and functionality of the photochemical apparatus, reflected in terms of functionality of PSII, as indicated by the higher values of F_*m*_ and F_*v*_/F_*m*_ after 30 days of bioaugmentation. Higher ETR values in inoculated *S. ramosissima* growing in polluted soil demonstrated the maintenance of the functionality of the electron transport chain, reinforcing the positive impact of PGPB inoculation. This fact may ensure enough electrons for carbon fixation through photochemical pathway, which is indeed reflected by A_*N*_ values under those suboptimal polluted conditions (Strasser et al., 2004).

Other physiological effects observed in this work were _*i*_WUE and water content augmentation in inoculated *S. ramosissima* in polluted sediments. _*i*_WUE is a well-established indicator of plant water managing under conditions of stress (Tardieu, 2012). In a similar manner, greater root growth in *S. ramosissima* inoculated with bacteria may aid to enhance water absorption capacity and, therefore, water content, iWUE and metal tolerance. In line with these results, previous works from authors of this study reported that bioaugmentation with indigenous PGPB incremented _*i*_WUE and other photosynthetic parameters like A_*N*_ and functionality of PSII in halophytes *S. densiflora*, *S. maritima*, and *Arthrocnemum macrostachyum* (Mateos-Naranjo et al., 2015; Mesa et al., 2015a,b; Navarro-Torre et al., 2017a,b; Paredes-Páliz et al., 2017; Mesa-Marín et al., 2019a).

Finally, our results revealed that *S. ramosissima* could be considered a heavy metal phytoaccumulator halophyte, as reflected by high concentrations of As (200 mg Kg^–1^), Cd (0.50 mg Kg^–1^), Cu (400 mg Kg^–1^), Ni (25 mg Kg^–1^), Pb (300 mg Kg^–1^), and Zn (300 mg Kg^–1^) recorded in roots of plants growing in polluted sediments (with approximately As 800 mg Kg^–1^, Cd 2 mg Kg^–1^, Cu 1000 mg Kg^–1^, Ni 30 mg Kg^–1^, Pb 1000 mg Kg^–1^, and Zn 800 mg Kg^–1^). Moreover, it is important to note that accumulation takes place mainly at root level. More specifically, root concentration of the analyzed metals in *S. ramosissima* growing in polluted Odiel sediments was approximately 20, 50, 20, 25, 30, and 15 times, respectively, higher than in leaves. Other authors also observed Cd accumulation capacity in this species (Pedro et al., 2013; Pérez-Romero et al., 2016). In the same line, As, Cd, Li, Ni, and Pb tolerance and accumulation have been noted by several authors for other *Salicornia* species, like *Salicornia brachiata*, *S. europaea*, and *Salicornia iranica* (Ozawa et al., 2010; Sharma et al., 2010; Kaviani et al., 2017a,b; Lou et al., 2020). Toxic metals are thought to enter root cells by means of the same uptake processes that move essential micronutrient metal ions, as for example competitive transport of Cd via voltage-gated cation channels. Also, these species seem to use different mechanisms for restricting the transport of toxic elements within the plant, including sub-cellular compartmentalization of the metal, namely in vacuoles, and the sequestration of the metal by specially produced organic compounds, like phytochelatins, concentrating metal in the plants roots (Pedro et al., 2013). Low metal translocation rates does not mean lower phytoremediation capacity, but different phytoremediation strategies. Thus, *S. ramosissima* may be appropriate for phytostabilization purposes, nor for phytoextraction. Bacteria inoculation with the selected consortium incremented heavy metal phytoaccumulation in *S. ramosissima*. However, we observed that this effect was not due to an amelioration of *S. ramosissima* accumulation ability, but to an increment in plant biomass, which permits a greater accumulation of heavy metals. This effect was also observed by Mesa et al. (2015a) after bioaugmentation with PGP endophytic bacteria in *S. maritima* growing in polluted Tinto salt marsh sediments. Nevertheless, other studies concluded that bioaugmentation with PGP rhizobacteria in halophytes *A. macrostachyum*, *Suaeda vera* and hyperaccumulators *S. maritima* and *S. densiflora* incremented significantly root metal phytoaccumulation ability (Mateos-Naranjo et al., 2015; Mesa et al., 2015b; Navarro-Torre et al., 2017a; Gómez-Garrido et al., 2018). Bacteria used in this work may increase plant tolerance through different mechanisms. For example, higher ETR values recorded in inoculated plants may suggest that several defense mechanisms could be activated, like the dissipation of energy excess as photorespiration or heat (Duarte et al., 2013), helping plants to reduce physiological stress when they are exposed to heavy metals. However, most of the energy absorbed would not take the photochemical pathway (Flexas et al., 2012). This fact affects photosynthetic productivity and, therefore, growth in plants not inoculated, which would support our experimental growth results. Also, PGPB may induce plant enzymatic antioxidant defense, as has been demonstrated by several authors (Kong et al., 2015; Mesa-Marín et al., 2018). Besides, PGPB may confer metal resistance by chemical detoxification, accumulation, transformation and sequestration of heavy metals, altering their phytoavailability in contaminated soils and thus diminishing the metal phytotoxicity (Kong and Glick, 2017). Moreover, numerous studies have suggested that inoculation of plants with PGPB that produce ACC deaminase and IAA, as is currently the case, may play an important role in improving metal phytoremediation by increasing heavy metal tolerance. However, most of these studies did not provide definitive and mechanistic proof of the direct involvement of these compounds (Kong and Glick, 2017). Indeed, there is a wide lack of knowledge regarding to specific mechanism underlying these effects, that opens a broad area of research.

A fact that is important to be noted is that PGPB bioaugmentation with this consortium did not promote in *S. ramosissima* higher accumulation rates of heavy metals in leaves, nor translocation from roots to leaves. This is relevant because *S. ramosissima* may be used as leafy vegetable (Ventura and Sagi, 2013; Ventura et al., 2015; Patel, 2016; Barreira et al., 2017) and it is may be considered a cash-crop halophyte in scenarios of climate change (Mesa-Marín et al., 2019c). In several studies, bacteria promoted metal translocation from roots to shoots, mainly through chemical transformation of metals that increment their bioavailability (reviewed in Kong and Glick, 2017). In this case, bacteria did not promote metal translocation to leaves. Lastly, a remarkable result was also that bioaugmentation increased Na concentration in leaves, as observed by other authors for the halophyte *A. macrostachyum* (Navarro-Torre et al., 2017a,b). This may be connected plant osmoregulation by salt uptake, as suggested previously by Redondo-Gómez et al. (2010).

As an approach for practical purpose, it would be interesting to study the effect of PGPB bioaugmentation over time. Important considerations are the survival and colonization potential of the inoculated strains, as well as the potential ecological risks of introducing non-native plant and microbial species into field sites. In this sense, it should be highlighted that rhizobacteria used as an inoculum in this experiment were previously isolated from *S. ramosissima* rhizosphere in SW Spain salt marshes. The rationale behind inoculation with native microorganisms isolated from the sampling site and plant is that a bacterial strain from a population that is spatially and temporally prevalent in a concrete habitat, has more chances to persist when reintroduced by inoculation, than others being alien or transient to such habitat, especially in polluted conditions like Odiel salt marsh (Vogel, 1996). Non-native inoculants may have poor survival and colonization ability, and therefore they may be not sufficient to effectively support phytoremediation over time. In a long period, it could be expected that inoculated bacteria successfully colonize the rhizosphere and effectively facilitate plant metal uptake over time (Ma et al., 2015), and even the microbial community diversity in the rhizosphere may decrease (Liu et al., 2015).

In conclusion, our data suggested that inoculation with heavy metal resistant PGPB improved *S. ramosissima* plant growth, photosynthetic fitness, intrinsic water use efficiency and water content in polluted sediments. Also, we observed that *S. ramosissima* was able to accumulate great concentrations of heavy metals, mostly at root level, and that bioaugmentation incremented *S. ramosissima* heavy metal phytoremediation potential due to plant biomass increment achieved after inoculation, which enabled a greater accumulation capacity. Thus, our results claim the potential of using inoculation with heavy metal resistant PGPB to strengthen the capacity of *S. ramosissima* as candidate species in phytoremediation of salty degraded ecosystems.

## Data Availability Statement

The original contributions presented in the study are included in the article/supplementary material, further inquiries can be directed to the corresponding author.

## Author Contributions

JM-M: conceptualization, methodology, formal analysis, writing – original draft, and writing – reviewing and editing. JP-R: methodology, formal analysis, and writing – reviewing and editing. SR-G: resources, funding acquisition, and writing – reviewing and editing. IR-L and EP: resources and writing – reviewing and editing. EM-N: methodology, formal analysis, resources, supervision, funding acquisition, and writing – reviewing and editing. All authors contributed to the article and approved the submitted version.

## Conflict of Interest

The authors declare that the research was conducted in the absence of any commercial or financial relationships that could be construed as a potential conflict of interest.
